# Machine Learning Methods Enable Predictive Modeling of Antibody Feature:Function Relationships in RV144 Vaccinees

**DOI:** 10.1371/journal.pcbi.1004185

**Published:** 2015-04-13

**Authors:** Ickwon Choi, Amy W. Chung, Todd J. Suscovich, Supachai Rerks-Ngarm, Punnee Pitisuttithum, Sorachai Nitayaphan, Jaranit Kaewkungwal, Robert J. O'Connell, Donald Francis, Merlin L. Robb, Nelson L. Michael, Jerome H. Kim, Galit Alter, Margaret E. Ackerman, Chris Bailey-Kellogg

**Affiliations:** 1 Department of Computer Science, Dartmouth College, Hanover, New Hampshire, United States of America; 2 Ragon Institute of Massachusetts General Hospital, Massachusetts Institute of Technology, and Harvard, Boston, Massachusetts, United States of America; 3 Department of Disease Control, Ministry of Public Health, Nonthaburi, Thailand; 4 Faculty of Tropical Medicine, Mahidol University, Bangkok, Thailand; 5 Armed Forces Research Institute of Medical Sciences, Bangkok, Thailand; 6 Department of Retrovirology, U.S. Army Medical Component, AFRIMS, Bangkok, Thailand; 7 Global Solutions for Infectious Diseases (GSID), South San Francisco, California, United States of America; 8 US Military HIV Research Program, Walter Reed Army Institute of Research, Silver Spring, Maryland, United States of America; 9 Henry Jackson Foundation HIV Program, US Military HIV Research Program, Bethesda, Maryland, United States of America; 10 Thayer School of Engineering, Dartmouth College, Hanover, New Hampshire, United States of America; Boston University School of Medicine, UNITED STATES

## Abstract

The adaptive immune response to vaccination or infection can lead to the production of specific antibodies to neutralize the pathogen or recruit innate immune effector cells for help. The non-neutralizing role of antibodies in stimulating effector cell responses may have been a key mechanism of the protection observed in the RV144 HIV vaccine trial. In an extensive investigation of a rich set of data collected from RV144 vaccine recipients, we here employ machine learning methods to identify and model associations between antibody features (IgG subclass and antigen specificity) and effector function activities (antibody dependent cellular phagocytosis, cellular cytotoxicity, and cytokine release). We demonstrate via cross-validation that classification and regression approaches can effectively use the antibody features to robustly predict qualitative and quantitative functional outcomes. This integration of antibody feature and function data within a machine learning framework provides a new, objective approach to discovering and assessing multivariate immune correlates.

## Author Summary

Antibodies are one of the central mechanisms that the human immune system uses to eliminate infection: an antibody can recognize a pathogen or infected cell using its Fab region while recruiting additional immune cells through its Fc that help destroy the offender. This mechanism may have been key to the reduced risk of infection observed among some of the vaccine recipients in the RV144 HIV vaccine trial. In order to gain insights into the properties of antibodies that support recruitment of effective functional responses, we developed and applied a machine learning-based framework to find and model associations among properties of antibodies and corresponding functional responses in a large set of data collected from RV144 vaccine recipients. We characterized specific important relationships between antibody properties and functional responses, and demonstrated that models trained to encapsulate relationships in some subjects were able to robustly predict the quality of the functional responses of other subjects. The ability to understand and build predictive models of these relationships is of general interest to studies of the antibody response to vaccination and infection, and may ultimately lead to the development of vaccines that will better steer the immune system to produce antibodies with beneficial activities.

## Introduction

Antibodies provide the correlate of protection for most vaccines [1]. This correlation is often thought to be mechanistic, as in numerous disease settings passively transferred antibodies provide protection from infection [2]. Yet, the fact that some vaccines that induce an antibody response do not provide protection indicates that beyond presence and prevalence, there are specific antibody features associated with protection: that is, not all antibodies are created equal. Efforts to develop a protective HIV vaccine may represent the setting in which the discrepancy between the generation of a robust humoral immune response and generation of protective humoral immunity has been most apparent. That this might be a more general observation is suggested by recent dengue vaccine trials, where protection was seen but did not appear to correlate with the well-established virus neutralization assay [3,4].

The significant challenges to inducing antibodies with potent anti-HIV activity have been well described [5]. Due to viral diversity, vaccine-specific antibodies may or may not recognize circulating viral strains [6]. Furthermore, beyond viral recognition, binding antibodies vary considerably in their ability to neutralize diverse viral variants (case studies in [7,8] and reviewed in [9]), with most antibodies possessing weak and/or narrow neutralization activity [10]. While generating broadly neutralizing antibodies represents a cornerstone of HIV vaccine efforts, as these antibodies clearly block infection in animal models [11], vaccines tested thus far have induced antibodies with only a limited ability to neutralize viral infectivity [12]. However, beyond this role in the direct blockade of viral entry, antibodies mediate a remarkable repertoire of protective activities through their ability to recruit the antiviral activity of innate immune effector cells. Yet, here as well, the ability of HIV-specific antibodies to act as molecular beacons to clear virus or virus-infected cells is also widely divergent [13].

Given the diversity of viral variants, the diversity of antibody binding and neutralization profiles driven by the IgG variable (Fv) domain, and the diversity of antibody effector activity driven by the IgG constant (Fc) domain, the landscape of antibody activity is perplexingly complex. While a number of structure:function relationships have been characterized in terms of virus recognition, neutralization, and innate immune recruiting capacity, our understanding of the relationship between antibody features and their protective functions remains incomplete. However, the recent development of high-throughput methods to assess properties of both antigen recognition and innate immune recognition [14] offers more fine-grained information about the antibody response, which could feed into the development of models to inform our understanding of antibody activity.

The moderate success of the RV144 HIV vaccine trial, in which partial protection from infection was observed [15], presents the opportunity to study antibody structure:function relationships in the first HIV vaccine to demonstrate efficacy. Importantly, within this trial, the correlates of reduced risk of infection were binding antibodies, and, in the absence of an IgA response, antibody function, in the form of natural killer (NK) cell-mediated antibody-dependent cellular cytotoxity [16]. Subsequent analysis has supported these findings: with evidence of the impact of variable domain-specific antibodies apparent in the sequences of breakthrough infections [17], and antibodies of the IgG3 subclass associated with reduced risk of infection [18]. Because the vaccine was partially efficacious, studying the diversity of antibody responses among volunteers has the potential to help identify novel immune correlates. Thus, this trial represents a compelling opportunity to profile antibody structure:function relationships from the standpoint of relevance to protection and an excellent setting in which to apply machine learning methods to characterize the relationship between antibody features and function in a population whose response to vaccination varied in a clinically relevant way.

Here, we study the relationships between biophysical data regarding HIV-specific antibodies induced by the RV144 vaccine regimen, and corresponding functional properties that have previously been correlated with better clinical outcomes in HIV infected subjects [19–21] as well as the protection observed in RV144. These effector functions are mediated by the combined ability of an antibody’s Fab to interact with the antigen and its Fc to interact with a set of FcR expressed on innate immune cells. Just as Fab variation impacts antigen recognition, Fc variation in IgG subclass dramatically influences FcR recognition, and antibody effector function is widely divergent among antibodies from different subject groups in ways that are not explained by titer, or the magnitude of the humoral response [22]. Therefore, we characterize the combination of antigen specificity and subclass in a multiplexed fashion (“antibody features”), and couple that characterization with assessments of effector activities from cell-based assays (“antibody functions”). This antibody feature and function data have previously been subjected to univariate correlation analysis, which identified associations between gp120-specific IgG3-subclass antibodies and coordinated functional responses in RV144 subjects. Conversely IgG2- and IgG4-subclass antibodies were associated with decreased activity, and subsequent depletion studies confirmed these discoveries [23].

In order to discover and model multivariate antibody feature: function relationships in data from RV144 vaccinees, we employ a representative set of different machine learning methodologies, within a cross-validation setting that assesses their ability to make predictions for subjects not used in model development. While “predict” often connotes prospective evaluation, here, as is standard in statistical machine learning, it means only that models are trained with data for some subjects and are subsequently applied to other subjects in order to forecast unknown quantities from known quantities. In particular, we show that not only are antibody features correlated with effector functions, but that computational models trained on feature: function relationships for some subjects can make predictions regarding the functional activities of other subjects based on their antibody features. Using unsupervised methods we find patterns of relationships between antibody features and effector functions as well as among features themselves. Then, using classification methods we demonstrate via cross-validation that antibody features support robust qualitative predictions of high vs. low function, and using regression methods we likewise demonstrate that the features can enable quantitative predictions of functionality across multiple, divergent activities. The various methodologies are relatively consistent in both performance and identified features, giving confidence in the general procedure and the information content in the data. This objective approach to developing predictive models based on patterns of antibody features provides a powerful new way to uncover and utilize novel structure:function relationships.

## Results

To model antibody feature-function relationships we analyzed samples from 100 subjects in the RV144 trial. A set of 3 different cell-based assays was conducted to characterize the functional activity of these samples, providing data regarding the effector function of antibodies induced by RV144 including: gp120-specific antibody dependent cellular phagocytosis (ADCP) by monocytes [24], antibody dependent cellular cytotoxicity (ADCC) by primary NK cells [25], and NK cell cytokine release (namely the combination of IFNγ, MIP-1β, and CD107a) [23]. Antibody features were assessed using a customized microsphere array [14] to characterize the antibodies induced by the vaccine in terms of their antigen specificity (gp140, gp120, V1V2, gp41, and p24) and IgG subclass (IgG1, IgG2, IgG3, and IgG4). For both the array-generated antibody feature data, and cell-based assay assessment of antibody functional activity, excellent discrimination between placebo (n = 20) and vaccinated (n = 80) subjects was observed [23]. The dataset is provided as a spreadsheet (S1 Dataset).

**Fig 1:**
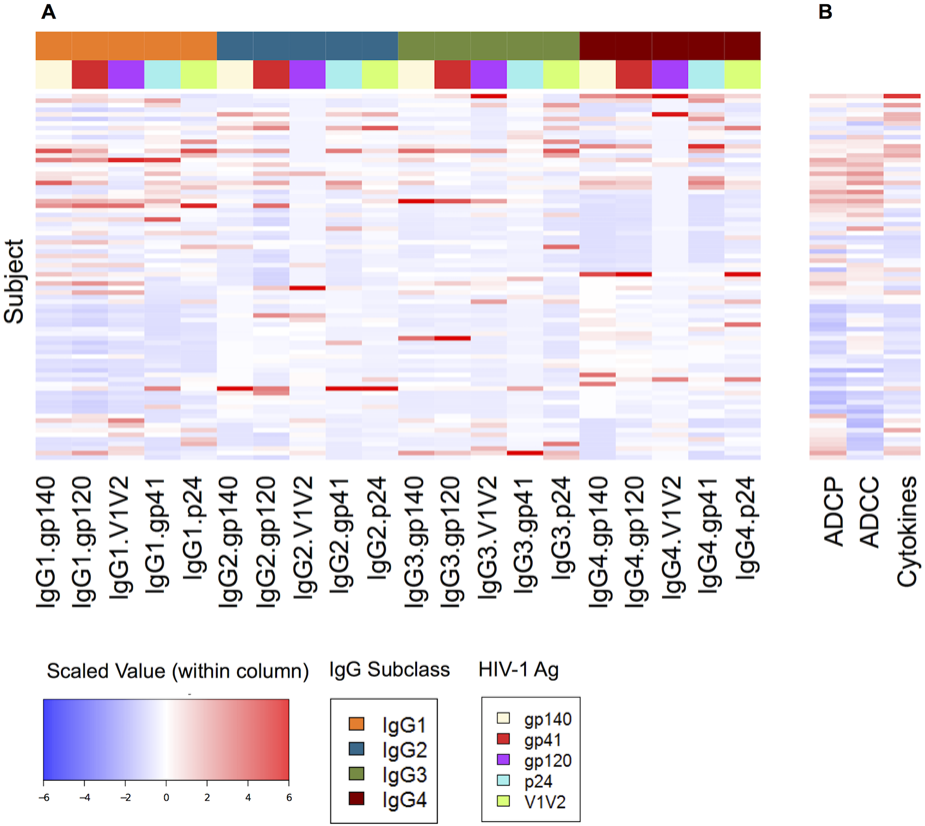
Input data. For each of 80 vaccinated subjects (rows), measurements of (A) 20 antibody features (4 IgG subclasses with 5 antigen specificities) and (B) 3 effector functions. The heatmap colors indicate relative values within each column, standardized to a mean of 0, a standard deviation of 1, and truncated at 6σ. Color blocks above the antibody feature columns indicate IgG subclass and antigen specificity.

Fig 1 illustrates scaled and centered data for each antibody feature (Fig 1A) and functional measurement (Fig 1B) for the 80 vaccinated subjects. We note that the subsequent analyses all use scaled and centered feature data, as the different features are on different and somewhat arbitrary scales according to bead set and detection reagent, and this standardization enables combination of the relative feature levels across these different scales. As a linear transformation, the standardization does not affect linear models, though the additional preprocessing truncation to 6σ has an appropriate impact on outliers. The function data are only standardized for this visualization, as the assay values are meaningful for interpreting predictions.

As discussed in the introduction, the data and correlation analyses have been previously presented [23]; we recapitulate the most relevant points here to lead into our machine learning approaches. We observe that the antibody features and functions are far from uniform. The relative functional responses differ by subject and by function, though a number of subjects exhibit relatively strong or weak responses in multiple functions. Likewise, relative antibody feature strength differs by subject and feature, and notably some subjects exhibit relatively strong responses across multiple antigen specificities for a given IgG subclass and/or strong responses across multiple subclasses for a given antigen specificity. Finally, there are relationships between the features and functions by subject, e.g., a group of subjects with strong ADCP and ADCC responses appear also to have strong feature characteristics. In order to better extract, assess, and utilize such observations, machine learning techniques were applied to provide models of the relationship between characteristics of HIV-specific antibodies induced by vaccination, and their functional activity.

### Unsupervised learning

**Fig 2:**
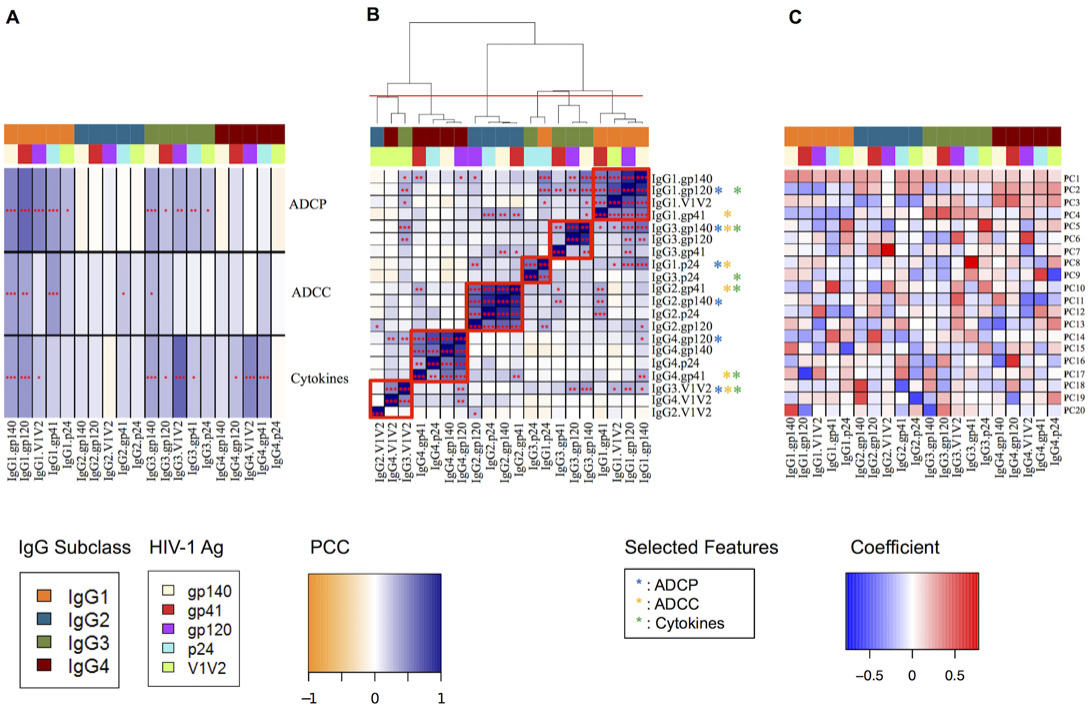
Unsupervised analysis of antibody features and functions. (A) Antibody feature:function correlations. IgG subclass and antigen specificity are indicated by color blocks. Cell colors indicate Pearson correlation coefficients (PCC), and p-values are represented by asterisks (* < = 0.05; ** < = 0.01; *** < = 0.001). (B) Feature:feature correlations, hierarchically clustered. Antibody feature color blocks, PCCs, and significances are denoted as in (A). Bisecting the dendrogram, as shown by the red line, results in 6 antigen.subclass clusters, each also denoted in the figure by a box. For each function, one feature was selected (starred: blue-ADCP; yellow-ADCC; green-cytokines) from each cluster to yield the filtered feature set. (C) Eigenvectors from principal component analysis. Cell colors indicate feature coefficients in the eigenvectors. Antibody feature color blocks are as in (A).

As Fig 2A illustrates, assessing antibody feature:function correlations across subjects enables the identification of several strong relationships. Consistent with their binding affinity to FcgR expressed on monocytes, IgG1 and IgG3 subclasses are most correlated with strong ADCP function, while IgG2 and IgG4 are less correlated or even mildly anticorrelated. Similarly, gp120 and V1V2 antigens tend to yield the strongest correlations, as would be expected given the direct experimental relevance of these antigens to this functional activity. For ADCC, the IgG1 correlations are weaker and the IgG3 correlations weaker still, while the IgG2 and IgG4 classes are now slightly more correlated (particularly IgG2.gp41). For the cytokines, strong IgG1 and IgG3 correlations are observed, particularly with gp120 and V1V2. The IgG4 subclass also yields some strong correlations, likely influenced by the large number of subjects with undetectable IgG4 responses (uniform colors within a column in Fig 1, no longer 0 after standardization), and rare subjects with strong IgG4 responses.

A number of antibody features exhibit similar patterns of correlation with function; these can largely be explained by correlations among the features themselves. Indeed, hierarchical clustering of the feature correlation profiles (Fig 2B) reveals that the features are not independent but in fact the true dimensionality of the data is lower than the number of original columns. The figure highlights six clusters of mutually correlated features formed by bisecting the dendrogram as indicated to strike a balance between the number of clusters and their visual coherence. An array of statistical methods to determine an optimal number of clusters gave substantially different answers from each other, though the optimal partitions they identified were largely consistent how one might manually divide the dendrogram (results not shown). Some of these clusters are defined by Ab subclass (each IgG subclass dominates one cluster), while others are defined by antigen specificity (V1V2 and p24 clusters are also observed). Correlations between IgG1 and IgG3-defined clusters are also observed. The combination of the feature:feature clustering and the feature:function correlations observed suggests that different groups of subjects produce characteristically different antibody responses, yielding different functional outcomes.

The strong relationships apparent among antibody features (indicating lower intrinsic dimensionality) likely result in redundancy in terms of their contributions to functional predictions. To support the supervised analysis below, a set of “filtered” feature sets was developed for each function. Filtered features were selected by choosing the feature most strongly correlated with the function within each cluster, in terms of the magnitude of the Pearson correlation coefficient (Fig 2A). Filtered features for each functional measurement are starred in Fig 2B, and span the full range of subclasses and antigen specificities. Thus, while redundancy is reduced, the ability to obtain insights into the relative contributions of each feature type to functional activities is maintained. While there are non-negligible correlations outside the clusters (and indeed between these selected features), the supervised results show that they have little impact on predictive performance.

As an alternative method to account for the possible redundancy among antibody features, a principal component analysis (PCA) was also performed. PCA yields a set of principal components (PCs) that represent the main patterns of variability of the antibody features across subjects. The PCs provide a new basis for the data; i.e., each observed feature profile is a weighted combination of the PC profiles, so we can think of the PCs as “eigen-antibodies”. In contrast to the filtered features, the principal components are composites, and by inspecting their composition, we can see the patterns of concerted variation of the underlying antibody features. Fig 2C illustrates the principal components and S1 Fig provides the corresponding eigenvalue spectrum (the relative amount of variance captured by each PC). While PC1 is essentially a constant offset by which to scale the overall magnitude of a feature profile, the other leading PCs reflect many of the same relationships also observed in the clustering analysis, including both subclass relationships and antigen specificity relationships. In particular, PC2 largely contrasts IgG2/4 vs. 1/3 composition, PC3 IgG4 vs. others, and PC4 IgG3 vs. others, while PC5 focuses on the relative p24-associated contribution, PC6 that of V1V2, and PC7 apparently an even finer-grained V1V2 specificity. As these leading seven principal components are the most readily interpretable and cover a large fraction of the variance in the data (S1 Fig), they are used for supervised learning below, and trailing PCs are dropped.

The unsupervised analysis suggests that there is indeed a high level of information content in the data, evidenced by the relationships among features identified by the clustering and PCA approaches, the correlations between the antibody features and the functions, and the agreement of these relationships with biological intuition. The strong relationships uncovered by these methods suggest that it might be possible to build models to predict functions from features, whether directly measured features or derived composites.

### Supervised learning: Classification

We first sought to robustly classify antibody function as high or low, relative to the median. To assess how much this discrimination depends on the classification approach utilized rather than the underlying information content in the data, we employed three different representative classification techniques: penalized logistic regression (a regularized generalized linear model based on Lasso), regularized random forest (a tree-based model), and support vector machine (a kernel-based model). Furthermore, in order to assess the effect of reducing redundancy and focusing on the most interpretable feature contributions, three different sets of input features were considered: the complete set (20 features: 4 subclasses * 5 antigens), the filtered set with one feature selected from each cluster based on correlation with function (6 features), and the PC features (7 leading PCs), as illustrated in Fig 2. Separate classifiers were built for each function and each input feature set.

**Fig 3:**
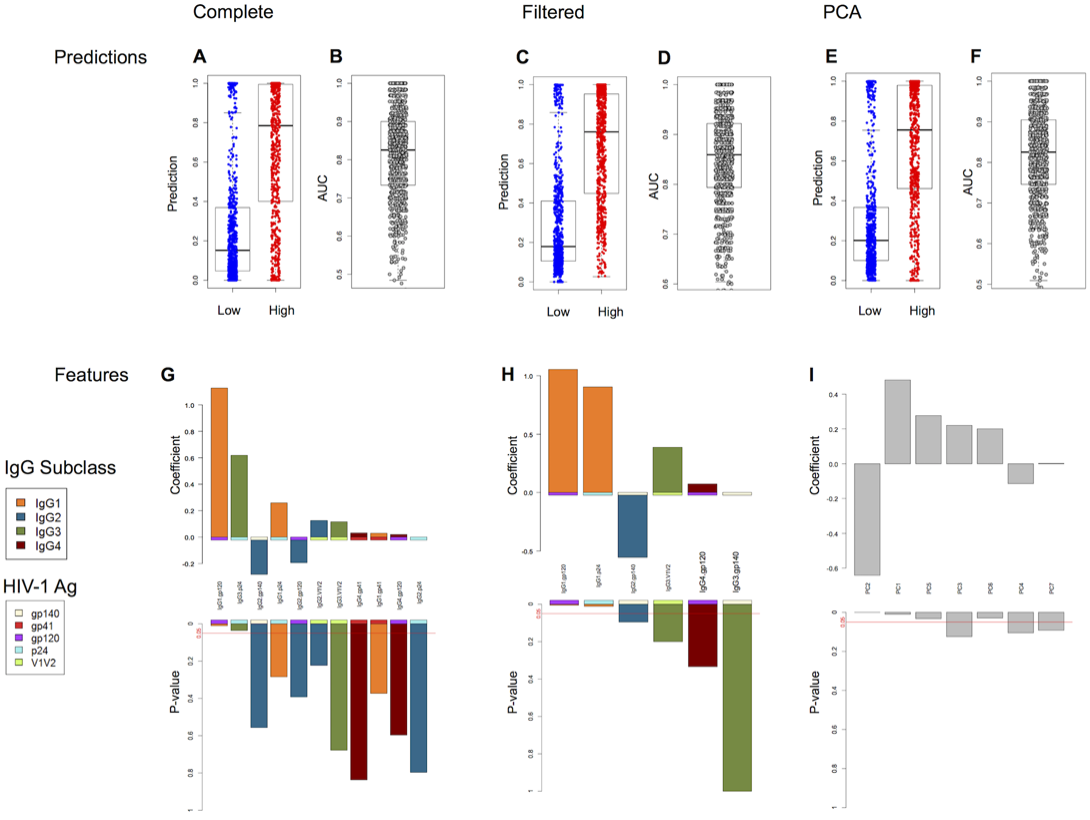
Classification of ADCP from antibody features by penalized logistic regression. (A-F) Prediction results by 200-replicate five-fold cross-validation, illustrating PLR values (>0.5 predicted high ADCP; <0.5 predicted low) for one replicate (A,C,E) and providing area under the ROC curve (AUC) over all 200 replicates (B,D,F). Box & whisker plots show the median (thick center line), upper and lower quartiles (box), and 1.5 times the interquartile range (whiskers); all points are also plotted in a jittered stripchart. Colors for the classification examples indicate high (red) and low (blue) observed ADCP. (G-I) Coefficients and p-values of the features for a model trained on all subjects. Different input features were used in classification: (A,B,G) the complete set; (C,D,H) the filtered set; (E,F,I) the principal components. Colors for the feature coefficients indicate antibody subclass and antigen-specificity. For convenience, a red line is drawn at p = 0.05.

Fig 3 summarizes the classification results for ADCP by penalized logistic regression. To assess the overall performance, we conducted 200 replicates of five-fold cross-validation. That is, for each of 200 replicates, the subjects were randomly partitioned into five equal-size sets, or “folds”, and five different models were constructed. Each model was trained using data for four of the sets of subjects, and then was used to make predictions for the fifth “held-out” set. The predictions for the held-out subjects were compared against the known (but ignored for training) values, and performance assessed accordingly. By repeating this 200 times, the impact of the random split can be factored out.

Fig 3A illustrates the predictions on one replicate (combining all five of its folds, with each serving separately as test data) and Fig 3B summarizes the resulting area-under-ROC-curve (AUC) over all 200 replicates (computing AUC only on test data). This data poses a difficult classification problem as there is not a clear distinction between high and low classes, which were simply defined by the median value. Nonetheless, even with a rigorous 200-replicate five-fold cross-validation, a mean AUC of 0.83 (standard deviation of 0.10) was observed, indicating that antibody features are highly and robustly predictive of high vs. low ADCP activity. Fig 3G shows the contributions of the antibody subclass-specificity features to a classifier trained on the whole dataset; while the coefficient values varied in individual folds, the same overall trends were observed over the different splits (results not shown).

Penalized logistic regression readily enables assessment of the relative importance of different features for classification. The model sums the feature values, each weighted by its specific coefficient, and then applies a logistic function to yield the predicted classification value. In order to counteract overfitting, the training process imposes a penalty relative to feature coefficients and thereby seeks a sparse model. The coefficients give the relative importance of each feature to the predictor; associated p-values indicate the confidence in those coefficient values (a large p-value indicates an unreliable estimate of the feature contribution). Thus we see, for example, that the two dominant and statistically significant (at an unadjusted 0.05 level) contributors to predicting ADCP class are IgG1.gp120 and IgG3.p24, capturing both key subclasses with two different antigen specificities. While not achieving statistically significant confidence in the coefficient value, negative contributions from IgG2 were also observed, consistent with the unsupervised analysis and the reduced ability of this subclass to bind to FcγR on phagocytes presumably due to blocking (i.e., preferred binding of antibodies with better affinity).

No systematic pattern was observed among the misclassified samples; they varied over the 200 splits and were distributed over the whole range of ADCP values. They did, however, tend to be those subjects with the weakest overall feature profiles, without large contributions from features with either positive or negative coefficients.

Despite penalization, a relatively large number of features contributed to the classifier, and to some extent they appeared redundant given the correlations among features observed in unsupervised analysis. To obtain a sparser and less redundant model, we trained classifiers using the filtered features from Fig 2B. Despite the reduction in data considered, Fig 3C and 3D shows that the resulting performance with the filtered feature set is comparable to that with the complete feature set, with a mean AUC of 0.84 (standard deviation 0.10). The feature contributions in Fig 3H are still driven by positive contributions of IgG1 and IgG3 with some of the same antigens, along with negative IgG2 (with gp140).

Though the goal of this study was not to comprehensively and rigorously assess feature selection methods, which would require further subsampling the data, we did investigate the sensitivity of the cluster-based filtering to our use of the features within each cluster that had the highest PCC. Thus we assessed each possible combination of features taken from the six clusters in Fig 2B. We found that on average an AUC of 0.79 was obtained, with a range from 0.67 to 0.87 and a standard deviation of 0.04 (recall that the PCC-based approach obtained 0.84). This result supports the conclusion that these groups of features do contain more or less redundant information in terms of predicting function. Using the best correlated features provides a sparse model that predicts as well as the model built from the complete feature set, and carries the advantage of being less likely to perform well due to overfitting, and thus more interpretable in terms of the underlying biology.

As noted above, PCA provides an alternative means commonly used to reduce redundancy. Thus we also trained classifiers using the principal components as features. Using these alternative, composite features, performance quality was maintained (Fig 3E and 3F), with a mean AUC of 0.82 (standard deviation 0.11). Inspecting the key PCs contributing to a classifier, we see that PC2 (IgG2/4 vs. 1/3) makes the biggest contribution, modulated by subclass contributions in PC3 (IgG4) and PC4 (IgG3) and antigen contributions in PC5 (p24), and PC6 (V1V2) (Fig 3I). Thus the PCA-based approach is largely consistent with the others, with subclass and antigen specificity again working in concert to predict function.

Table 1 summarizes the classification performance under all three classification methods. All three machine learning techniques perform quite well, despite the difficulty of the median-split classification problem and the rigorous five-fold cross-validation assessment. The PLR model is consistently a bit better, and performance is essentially equivalent for each technique across the different feature sets (complete, filtered, or PC), suggesting that over a wide range of different modeling approaches, antibody features are indeed robustly predictive of qualitative effector function.

**Table 1:** Summary of classification (shaded) and regression (unshaded) performance for prediction of antibody function across different input features and multiple learning techniques.

Function	Prediction Assessment*	Method	Input features		
			Complete	Filtered	PCs
ADCP	Classification	PLR	0.83 (0.10)	0.84 (0.10)	0.82 (0.11)
		RRF	0.77 (0.63)	0.79 (0.07)	0.79 (0.07)
		SVM (Radial)	0.71 (0.07)	0.73 (0.06)	0.70 (0.07)
	Regression	Lars	0.64 (0.15)	0.61 (0.15)	0.61 (0.15)
		GP (Poly)	0.56 (0.18)	0.53 (0.16)	0.55 (0.16)
		SVR (Radial)	0.58 (0.16)	0.56 (0.19)	0.58 (0.15)
ADCC	Classification	PLR	0.73 (0.12)	0.74 (0.12)	0.70 (0.13)
		RRF	0.65 (0.07)	0.62 (0.07)	0.63 (0.06)
		SVM (Radial)	0.60 (0.06)	0.56 (0.06)	0.60 (0.06)
	Regression	Lars	0.40 (0.18)	0.42 (0.18)	0.36 (0.20)
		GP (Poly)	0.13 (0.21)	0.24 (0.21)	0.23 (0.20)
		SVR (Radial)	0.32 (0.19)	0.14 (0.24)	0.20 (0.21)
Cytokines	Classification	PLR	0.80 (0.11)	0.76 (0.11)	0.80 (0.11)
		RRF	0.75 (0.06)	0.74 (0.06)	0.72 (0.07)
		SVM (Radial)	0.67 (0.07)	0.67 (0.06)	0.65 (0.07)
	Regression	Lars	0.58 (0.20)	0.51 (0.21)	0.44 (0.21)
		GP (Poly)	0.43 (0.27)	0.46 (0.24)	0.48 (0.25)
		SVR (Radial)	0.40 (0.18)	0.55 (0.15)	0.43 (0.18)

* For classification: AUC (standard deviation); for regression: PCC (standard deviation).

Corresponding classifiers were also built for ADCC and cytokine profiles using each of the three different learning techniques and three different feature sets; the performance of these models is also summarized in Table 1. The cytokine classifiers perform nearly as well as the ADCP ones, and the ADCC classifiers less accurately but still strikingly well. The choice of feature set (complete, filtered, PC) did not have a substantial effect on performance. The PLR approach was generally superior, with RRF quite comparable and SVM somewhat degraded but still yielding good performance. Thus our hypothesis that antibody features enable robust, high-quality prediction of antibody function is well-supported by the summary results for each of three distinct effector functions. Furthermore, the logistic regression model enables straightforward identification of the key contributors, and points toward feature roles consistent with known IgG and innate immune cell biology.

S2 Fig (ADCC) and S3 Fig (cytokines) detail the PLR results. For ADCC, the key contribution using the complete feature set is made by IgG1.gp41, consistent with ADCP in terms of subclass, but driven by a different antigen. In contrast there appears to be less contribution from IgG3 and IgG4 contributes positively (though the confidence in that coefficient is lower). Several of the selected features are gp41-specific. These trends are also largely reflected in the unsupervised feature:function correlations in Fig 2A. The cytokine feature usage is driven by IgG1 and IgG3 (with different antigens), along with an inconsistent contribution from IgG4, negative with p24 and gp140 and positive with gp41. Since these features are themselves highly correlated (Fig 2C), it appears that, despite the penalization in the PLR approach, this model is likely to be overfit. For both functions, feature filtering results in much the same relative contributions as for the complete feature set, with coefficients more strongly focused on a few key features. Notably, the inconsistent use of IgG4 features is eliminated by filtering. The ADCC response for the PC features is driven by PC6, which appears primarily to distinguish the V1V2-specificity. The PC features selected for the cytokines are more consistent with the other feature sets, with PC2 (IgG2/4 vs. 1/3) modulated by PC6 (V1V2), along with an IgG4.V1V2 down-selection via PC7.

The median-based dichotomization into high and low classes allowed us to characterize which antibody features were generally associated with superior effector function, but the division between high and low was quite fuzzy, with many subjects on the border. Thus we also performed classification into the top and bottom quartiles (ignoring the middle half). While unsurprisingly, the best vs. worst classification performance was better than the better vs. worse, our focus was the features driving class assignment, which remained largely consistent (results not shown). In particular, IgG1, with a variety of antigens, was the dominating contributor, often complemented by an IgG3-based feature; in addition, IgG4 features contributed negatively to ADCP but positively to the other two functions.

### Supervised learning: Regression

Given the quality of the classification results, both in predictive ability and in terms of clear and consistent use of biologically significant features, we sought to build quantitative models to predict function. Again, three representative techniques were used to broadly assess the general ability of the data to support predictive models: Lars (regularized linear regression based on Lasso), Gaussian process regression (a nonlinear model), and support vector regression (a kernel-based model). We again built separate models for each function, under each set of input features.

**Fig 4:**
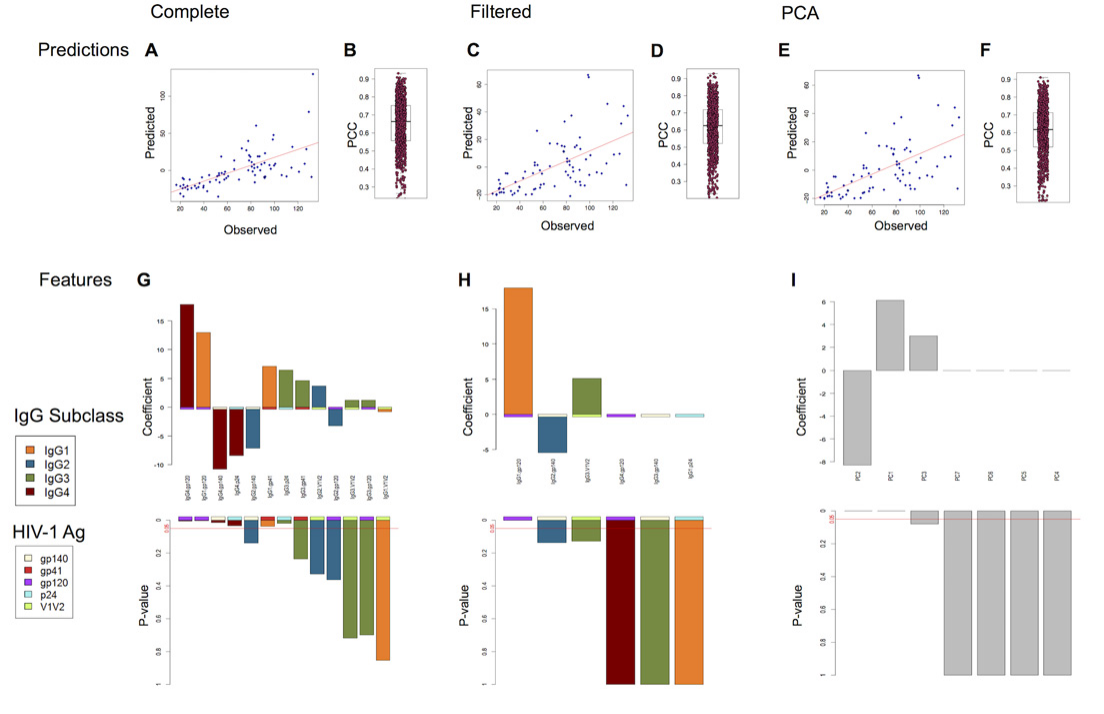
Regression modeling of ADCP from antibody features by Lars. (A,C,E) Representative regression scatterplot based on leave-one-out cross-validation, (B,D,F) PCCs for 200-replicate five-fold cross-validation. (G-I) Coefficients and p-values of the features for a model trained on all subjects. Different input features were used: (A,B,G) the complete set; (C,D,H) the filtered set; (E,F,I) the principal components. Box & whisker plots show the median (thick center line), upper and lower quartiles (box), and 1.5 times the interquartile range (whiskers); all points are also plotted in a jittered stripchart. Colors for the feature coefficients indicate antibody subclass and antigen-specificity.

Fig 4 summarizes the ADCP regression results from Lars across the complete feature set (Fig 4A, 4B and 4G), the filtered features (Fig 4C, 4D and 4H), and PCs (Fig 4E, 4F and 4I). While 200-replicate five-fold cross-validation was used for performance assessment, leave-one-out cross-validation (LOOCV) was used to generate representative scatterplots of experimental vs. predicted functional values, as is appropriate when viewing LOOCV as a form of jackknife. The models are clearly predictive of ADCP, obtaining a mean Pearson correlation coefficient PCC = 0.64 (standard deviation 0.15) over the 200-replicate five-fold. An example LOOCV scatterplot is illustrated in Fig 4A; the correlated trend between observed and predicted ADCP is clear. Notably, the LOOCV and five-fold PCCs (Fig 4B) were similar.

As a form of linear regression, Lars enables direct inspection of the coefficients contributing to the prediction. As with penalized logistic regression, the regularization employed by Lars in training seeks to force coefficients to zero and yield a sparse model. Fig 4G depicts the coefficients and their p-values for a model trained on the entire set of features. Among the largest and most-confident coefficients, we see that IgG1.gp120 is again a strong positive contributor, joined by the related IgG1.gp41 and IgG3.p24, and IgG2.gp140 is a strong negative contributor. Despite the Lars penalization, the model incorporates offsetting positive and negative contributions from IgG4 under different antigens, though these features are highly correlated with each other (Fig 2C).

In inspecting outliers, we found that the most overpredicted subjects (i.e., predicted ADCP much larger than experimental) were characterized by a relatively large number of features with large values. A possible statistical explanation for this is that the model works best when a few features are indicative of the response. A possible experimental explanation is that there are competitive effects, and indeed the contributions from multiple good antibodies are not additive in terms of recruiting effector cells.

As with classification, we sought to focus on the most informative and non-redundant features in order to reduce the risk of overfitting and develop more readily interpretable models. Models learned from the filtered features from Fig 2B maintain about the same accuracy (mean PCC = 0.61 with standard deviation 0.15 for the 200-replicate five-fold (Fig 4D); an example LOOCV scatterplot is illustrated in Fig 4C). By inspecting features for a model trained on the filtered features (Fig 4H), we see that the prediction is driven primarily by IgG1.gp120 and IgG3.V1V2, with a negative contribution from IgG2.gp140. The contradictory IgG4 contribution is resolved. Similarly, PCA-based models attain mean PCC of 0.61 with standard deviation 0.15 (Fig 4E and 4F), based largely on PC2 (IgG2/4 vs. 1/3) and somewhat on PC3 (IgG4 vs. others), as can be seen in Fig 4I.

The performance of all three machine learning methods using all three feature sets is summarized in Table 1. As with classification, the linear model dominates, and all methods perform similarly well with any of the input feature sets.

Lars-based regression results for ADCC and cytokines are presented in S4 Fig, and S5 Fig, respectively, and summarized in Table 1. While providing the desired trend overall (with a few striking outliers), the ADCC regression with the complete feature set does not have as high a PCC (mean 0.40, standard deviation 0.18) as the ADCP one (mean 0.64, standard deviation 0.15). With a mean PCC of 0.58 and a standard deviation of 0.20, the cytokine regression is comparable to that observed in predicting ADCP, though the representative scatterplot is not as pleasing to the eye due to the density of subjects with low values. Feature filtering achieves essentially the same performance for ADCC but a degradation in the cytokine performance as assessed by PCC, though the scatterplot appears roughly as good. The switch to PC features degrades the PCC measurements for both functions, though again yielding trends that appear satisfactory visually.

As for classification, ADCC prediction is driven by IgG1.gp41, with IgG1.gp140 also contributing strongly, and probably redundantly, as suggested by Fig 2B. As we saw for classification, the cytokine model has positive IgG1 and IgG3 contributions and inconsistent IgG4 contributions. For the filtered features, the ADCC model is focused on IgG1.gp41, with IgG1.gp140 replaced by the related IgG3.gp140. The feature-filtered model for cytokines retains IgG3.V1V2 and IgG1.gp120 contributions and resolves the IgG4 inconsistency, leaving a positive IgG4.gp41 contribution as observed in Fig 2A. When switching to the PCA-derived features, the ADCC regression model is driven by PC6 (V1V2), as with the classification model, while the cytokine regression model agrees with the classification model in its use of PC6 and PC7 with opposing signs, while weakening PC2 (IgG2/4 vs. 1/3) perhaps in lieu of added contributions from PC4 (IgG4) and PC3 (IgG3).

Table 1 summarizes the performance for ADCC and cytokines under all machine learning techniques and feature sets. Once again the linear model dominates the nonlinear models, particularly for ADCC. With the complete feature set, this is likely directly attributable to overfitting, and an improvement of the nonlinear methods upon starting with the filtered features though not as much with the PC features, was observed. As discussed in the methods, the presented results employ a polynomial kernel for Gaussian Process Regression and a radial basis kernel for Support Vector Regression; alternative kernels did not improve the performance. While the disappointing performance of the more sophisticated methods could potentially be improved by custom feature selection methods or parameter tuning, our goal here is not to provide such a benchmark but rather to establish the general scheme of predictive modeling of antibody feature: function relationships. The overall concordance observed between different feature sets, different regression and classification methods, and across multiple, complex, antibody functional activities, subjected to cross-validation assessment, demonstrates that indeed antibody features can be used to effectively predict functional activities.

## Discussion

We have demonstrated that the integration of antibody feature and function data via machine learning models and methods helps identify and make use of critical landmarks in the complex landscape of antibody feature:function activity. Sets of features emerge from patterns in the data, and these feature sets are able to robustly predict high/low levels of function, and are even informative enough to support quantitative predictions of functional activity. The subclass-specific contributions observed here are consistent with expectations, according to the receptors on the relevant effector cells, and the activity profiles among IgG subclasses [26]. At the same time, the approach provides a finer resolution picture of the interrelationships among antigen specificity, subclass, and effector function.

In the case of RV144, it is worth noting that the vaccine included two different components, priming with canarypox ALVAC-HIV (vCP1521) and boosting with recombinant gp120 AIDSVAX B/E protein. Thus while the prime included the gp120, gp41, and p24 antigens evaluated here, the boost only included gp120. Furthermore, cell-based functional assays employed particular antigens to stimulate a response, and those studied here are gp120-specific. Thus we might expect to see differences within functional responses among subjects according to different overall specificities of their antibodies, or even within antibody specificities depending on whether they were raised in the setting of the prime or the boost. Accordingly, associations observed here, such as those between gp41-specific antibodies and functional activity in assays in which only gp120 is presented, clearly do not have mechanistic significance with respect to functional assays that characterize only gp120-specific responses. However, they may nonetheless provide useful associative markers that functionally differentiate overall antibody responses to priming and boosting or among subjects that were more finely grained than subclass and antigen-specificity alone.

The machine learning approaches employed here contrast with typical univariate correlation analysis in two important ways: simultaneously combining and down-selecting features, and assessing generalization performance in a predictive setting. These approaches incorporate multiple features into a model, but do so in a way that avoids simply “memorizing” artifacts of the samples, as is easily possible with a sufficient number of features for a small sample set. Cross-validation analysis then ensures that the models are not overfit, by testing how well predictions from a model trained on one set of data match observations for another set. This predictive assessment stands in contrast to typical correlation analysis, which uses all the data and simply evaluates quality of fit.

Redundancy among features confounds the interpretation of multivariate feature:function relationships. To account for redundancy, we have used representative, common approaches including feature selection within the learning algorithm (via regularization), feature filtering (via feature clustering), and feature combination (via principal components analysis). The approaches were all fairly comparable in performance for this dataset, perhaps due to the relatively small number of initial features. Larger feature sets may result in more substantial differences, and require additional techniques to reduce the number of features contributing to a model down from a highly redundant input set to a reduced but representative and robust set. For example, elastic net type approaches [27] might strike a beneficial balance between eliminating redundant features and averaging them out to improve robustness.

The goal of this paper is to demonstrate that it is possible to develop models able to robustly predict the broad functional activities of antibodies from data regarding antigen specificity and Fc characteristics, with an aim ultimately in developing models that will correlate with protection or risk of infection. Several representative methods were demonstrated, though a rigorous benchmarking comparison was not performed as that would require a larger, more diverse dataset. We conclude that while there are some clear differences in performance among the methods, they all show that there is sufficient information in the features to predictively model function. The penalized generalized linear models are generally very good, and provide the added advantage of easy interpretation and relatively low model complexity; as noted in the previous paragraph, a softer regularization might be beneficial in the future.

The relationships identified by machine learning methods can be used to drive prospective studies to test particular hypotheses regarding how particular antigen specificities and subclasses contribute to the stimulation of effector response. As an illustration, we note that subsequent to our modeling and characterization of feature:function relationships in the RV144 data, depletion studies confirmed a mechanistic role for antibodies associated with prediction quality. These experimental observations demonstrated that indeed IgG3 is important for a strong phagocytic response, with IgG3-depleted samples having significantly reduced ADCP activity [23]. Similarly, our models predicted that IgG4 has a negative impact on functional level, and an analogous depletion experiment did exhibit this trend across 2 different vaccine regimens, although the increase in activity in the RV144 samples when IgG4 was depleted did not meet statistical significance [23].

Due to the evident importance of innate immune recruiting for the protection observed in the RV144 trial, and given the unprecedented feature and function data available for a set of subjects from that trial, we have focused here on specific relationships within the repertoire of antibodies induced by this vaccine. However, the approach described here can also be productively applied in other settings, shedding light on relationships specific to particular cohorts, as well as different vaccination and infection contexts. By integrating diverse datasets, it may even be possible to uncover more general rules governing the ways that antibodies bridge the adaptive and innate arms, and how those rules can then be specialized in a context-dependent fashion.

While the present study demonstrated the ability of antibody features to predict functional activities, the longer-term goal is to predict the impact of vaccination. To this end, an important next step is a case/control study with the potential to tease apart signatures leading to protection. Even in the context of the functions assayed here, a more complex multi-output model could be built in order to ascertain signatures of desirable polyfunctional responses. The fact that some functions were better predicted than others in the models described here, may indicate that additional antibody feature information could contribute to improved model performance. In particular, ADCC activity, the function predicted most poorly by the antigen and subclass data used here, is known to be dependent on antibody glycosylation state [22], which was not assessed in this study. Feature data could be extended to characterize a wider range of relevant antibody features, including additional antigen specificities as well as characteristics of the Fc glycan structure, or interactions with the cellular antibody receptors expressed by NK cells and phagocytes.

Overall, we find that the parallel assessment of antibody function and antibody features can provide for development of models enabling quantitative predictions of functional activity across multiple, divergent antibody activities. Because these antibody functions have been associated with better clinical outcomes in HIV infected subjects, as well as the protection observed in RV144 and in many settings beyond HIV infection, but are poorly predicted by antibody titer, we anticipate that this type of predictive model can provide significant value, both in terms of permitting the substitution of high-throughput biophysical characterization for low-throughput cell-based assays, as well as for uncovering novel structure:function relationships that can inform vaccine design efforts.

## Methods

### Data collection and preprocessing

Plasma samples, provided by the MHRP and RV144 study group, were obtained from 100 participants in the RV144 vaccine trial [15], consisting of 20 placebo and 80 vaccinated subjects at week 26. Experimental methods used have been previously described [23]. Briefly, IgG was purified from all samples using Melon Gel according to the manufacturer’s instructions (Thermo Scientific). The functional activity of HIV-specific antibodies was determined in 3 different cell-based assays. Phagocytic activity was assessed using a monocyte-based assay in which the uptake of gp120-coated fluorescent beads is determined by flow cytometry [24]. Antibodies were tested at a concentration of 25 ug/ml MN. Similarly, the cytotoxicity profile of antibodies was tested at a concentration of 100 ug/ml in the rapid fluorescent ADCC assay, which assesses the ability of antibodies to drive primary NK cells to lyse gp120-pulsed target cells [25]. Lastly, NK cell degranulation and cytokine secretion were monitored by flow cytometry as described [23]. Surface expression of CD107a, and intracellular production of IFN-γ and MIP-1β were assessed, and the fraction of NK cells which were triple positive was determined. In order to profile antibody features, a customized antigen microsphere array was used to assess antibody specificity (gp120, gp140, V1V2, gp41, and p24) and subclass (IgG1,2,3,4) [14].

Array measurements for the vaccinees were standardized individually for each antigen.subclass feature as follows. Background signal level was derived from the values for that feature among placebos, as the placebo mean plus one standard deviation. This background was subtracted from each vaccinee. Finally, the vaccinee values for the feature were scaled and centered to a mean of 0 and a standard deviation of 1, with values truncated to 6σ.

For functional assays, data was not placebo-subtracted, but was instead inspected to ensure that low activity was observed in samples from placebo subjects

### Unsupervised learning

Antibody feature:function and feature:feature correlations were computed over the set of 80 vaccinated subjects and assessed using Pearson correlation coefficient and p-value.

Features were clustered based on the profile of their correlation coefficients over the set of all features. Hierarchical clusters were generated by the Ward linkage algorithm [28], assessing pairwise similarity between profiles in terms of Pearson correlation coefficient (i.e., 1-*r* dissimilarity). By visual inspection, six groups were identified in the resulting dendrogram. The R package NbClust was also used to assess optimal numbers of clusters according to a number of different indices [29]. For each function and each group, the feature with the largest-magnitude feature:function correlation coefficient was identified; each such feature also had the best feature:function p-value within its group, < = 0.001.

Principal component analysis was performed on the feature:subject data matrix (after preprocessing). Singular value decomposition was employed to determine a set of eigenvectors and corresponding eigenvalues, with the eigenvectors serving as a basis transformation matrix containing principal components that are linear combinations of the original features, and the eigenvalues indicating the amount of variance in the data captured by their eigenvectors. The top 7 were chosen for further use in supervised methods, by visual inspection of their components and their eigenvalues.

### Supervised learning: Classification

Three different and representative classification methods were employed: L_1_ penalized logistic regression (PLR) [30], regularized random forest (RRF) [31], and support vector machine (SVM) [32,33].

**PLR** is a form of logistic regression incorporating into the model evaluation a lasso penalty term λ||*β*||_1_, where *λ* is a tuning parameter and ||*β*||_1_ is the L_1_ norm of a coefficient parameter vector,*β*. Thus the learning favors sparse models, as zero-valued coefficients do not contribute to the penalty term. The R package “penalized” was used for PLR. It employs a greedy search to determine the best value for *λ* according to nested cross-validation (i.e., given a training set, doing an internal cross-validation within it to determine the performance under possible *λ* choices).

**RRF** is a decision tree-based method that generates multiple decision trees over bootstrap replicates of the data (i.e., a random forest), at each split selecting a feature from a randomly-sampled set based on an Gini index assessment of node impurity augmented with a regularization penalty to prefer a sparser set of selected features. The R package “RRF” was used for RRF-based learning. Two parameters were specified: mtry, the number of features to be randomly sampled at each split, which was set to the number of input features; and ntree, the number of trees or bootstrap samples, which was set to 2000 to obtain more reliable results. The regularization parameter is handled automatically by the method, based on the scores from a 0-penalty model.

**SVM** is a kernel-based nonlinear classifier that finds a separating hyperplane (in a space defined by the kernel) between the classes, so as to minimize the risk of classification error. The R package “e1071”, based on the C classification method of the libsvm library [34], was used for SVM-based classification. The standard linear, polynomial, and radial basis kernels were evaluated, and results presented for the radial basis function.

Default parameter values were used except where noted.

Each method was trained separately for each function with each of three different feature sets: the complete preprocessed set, the filtered set from the feature:feature clustering, and the set of principal components. To study the impact of selecting different features in the cluster-based filtering, the Lars method was also applied to each possible set of features combining one from each cluster.

To obtain robust characterization of classification performance, 200 replicate five-fold cross validation was employed; i.e., the data was randomly split into fifths, four used for training and one for testing, with 200 different such training/testing runs. The R package “ROCR” was used to calculate a cut-off independent evaluation of the area under the ROC curve (AUC) for each replicate.

To gain insights into the features driving the PLR classification performance, a model was also built using all subjects in order to obtain the best confidence in the coefficients.

In order to evaluate the impact (both prediction quality and feature usage) of median-based dichotomization, the PLR-based approach was applied in the same manner to a dataset limited to the subjects with the top and bottom quartile ADCP values.

### Supervised learning: Regression

Diverse representative approaches employed for regression were Lars [35,36], Gaussian Process Regression (GP) [37], and Support Vector Regression (SVR) [38].

**Lars** performs penalized linear regression with the L_1_-norm lasso penalty discussed above for PLR. The R package “parcor” was used for Lars. As with PLR the penalty weight was selected by cross-validation. The parameter for the number of splits was set to 10 for robust fitting.

**GP** performs nonlinear regression based on a stochastic process specified in terms of mean and covariance functions. Observed values are used to fit the functions and thereby predict unobserved ones. The R package “kernlab” was used for GP. A polynomial kernel function was used to fit the GP model, as it performed better than other kernels.

**SVR** is based on the same theory as SVM, discussed above, but uses the kernel-based approach to fit a regression model to reduce the quantitative prediction error. The R package “kernlab” was also used for SVR. As with SVM, we evaluated the standard linear, polynomial, and radial basis kernels and presented the results for the radial basis function.

Default parameter values were used except where noted.

The different feature sets were tested as described in the classification section.

Performance was assessed by Pearson correlation coefficient (PCC), *r*, between observed and predicted function value; *r* assesses the linear correlation (between -1 for perfectly anticorrelated and +1 for perfectly correlated), while *r* ^2^ represents the fraction of the variation explained. The PCC was computed over 200-replicate five-fold cross-validation. In addition, leave-one-out cross-validation was performed in order to generate representative scatterplots.

A Lars model was trained on all subjects in order to enable inspection of feature coefficients.

## Supporting Information

S1 Fig Principal component analysis eigenvalue plot. (A) Relative variance and (B) log absolute variance captured by each principal component. Red lines indicate truncation after the 7 leading principal components, which capture most of the variance and are most readily interpretable.
(TIF)

S2 Fig Classification of ADCC from antibody features by penalized logistic regression. (A-F) Prediction results by 200-replicate five-fold cross-validation, illustrating PLR values (>0.5 predicted high ADCP; <0.5 predicted low) for one replicate (A,C,E) and providing area under the ROC curve (AUC) over all 200 replicates (B,D,F). Box & whisker plots show the median (thick center line), upper and lower quartiles (box), and 1.5 times the interquartile range (whiskers); all points are also plotted in a jittered stripchart. Colors for the classification examples indicate high (red) and low (blue) observed ADCP. (G-I) Coefficients and p-values of the features for a model trained on all subjects. Different input features were used in classification: (A,B,G) the complete set; (C,D,H) the filtered set; (E,F,I) the principal components. Colors for the feature coefficients indicate antibody subclass and antigen-specificity. For convenience, a red line is drawn at p = 0.05.
(TIF)

S3 Fig Classification of cytokine release from antibody features by penalized logistic regression. (A-F) Prediction results by 200-replicate five-fold cross-validation, illustrating PLR values (>0.5 predicted high ADCP; <0.5 predicted low) for one replicate (A,C,E) and providing area under the ROC curve (AUC) over all 200 replicates (B,D,F). Box & whisker plots show the median (thick center line), upper and lower quartiles (box), and 1.5 times the interquartile range (whiskers); all points are also plotted in a jittered stripchart. Colors for the classification examples indicate high (red) and low (blue) observed ADCP. (G-I) Coefficients and p-values of the features for a model trained on all subjects. Different input features were used in classification: (A,B,G) the complete set; (C,D,H) the filtered set; (E,F,I) the principal components. Colors for the feature coefficients indicate antibody subclass and antigen-specificity. For convenience, a red line is drawn at p = 0.05.
(TIF)

S4 Fig Regression modeling of ADCP from antibody features by Lars. (A-F) Representative regression scatterplot based on leave-one-out cross-validation (A,C,E), and PCCs for 200-replicate five-fold cross-validation (B,D,F). (G-I) Coefficients and p-values of the features for a model trained on all subjects. Different input features were used: (A,B<G) the complete set; (C,D,H) the filtered set; (E,F,I) the principal components. Box & whisker plots show the median (thick center line), upper and lower quartiles (box), and 1.5 times the interquartile range (whiskers); all points are also plotted in a jittered stripchart. Colors for the feature coefficients indicate antibody subclass and antigen-specificity.
(TIF)

S5 Fig Regression modeling of cytokine release from antibody features by Lars. (A-F) Representative regression scatterplot based on leave-one-out cross-validation (A,C,E), and PCCs for 200-replicate five-fold cross-validation (B,D,F). (G-I) Coefficients and p-values of the features for a model trained on all subjects. Different input features were used: (A,B,G) the complete set; (C,D,H) the filtered set; (E,F,I) the principal components. Box & whisker plots show the median (thick center line), upper and lower quartiles (box), and 1.5 times the interquartile range (whiskers); all points are also plotted in a jittered stripchart. Colors for the feature coefficients indicate antibody subclass and antigen-specificity.
(TIF)

S1 Dataset Compiled antibody feature and function data [23]. (CSV)
