# Supplementary figures and images for: Machine Learning Methods Enable Predictive Modeling of Antibody Feature:Function Relationships in RV144 Vaccinees
Source: PLoS Comput Biol. 2015 Apr 13;11(4):e1004185. doi: 10.1371/journal.pcbi.1004185 (PMC4395155; doi:10.1371/journal.pcbi.1004185)

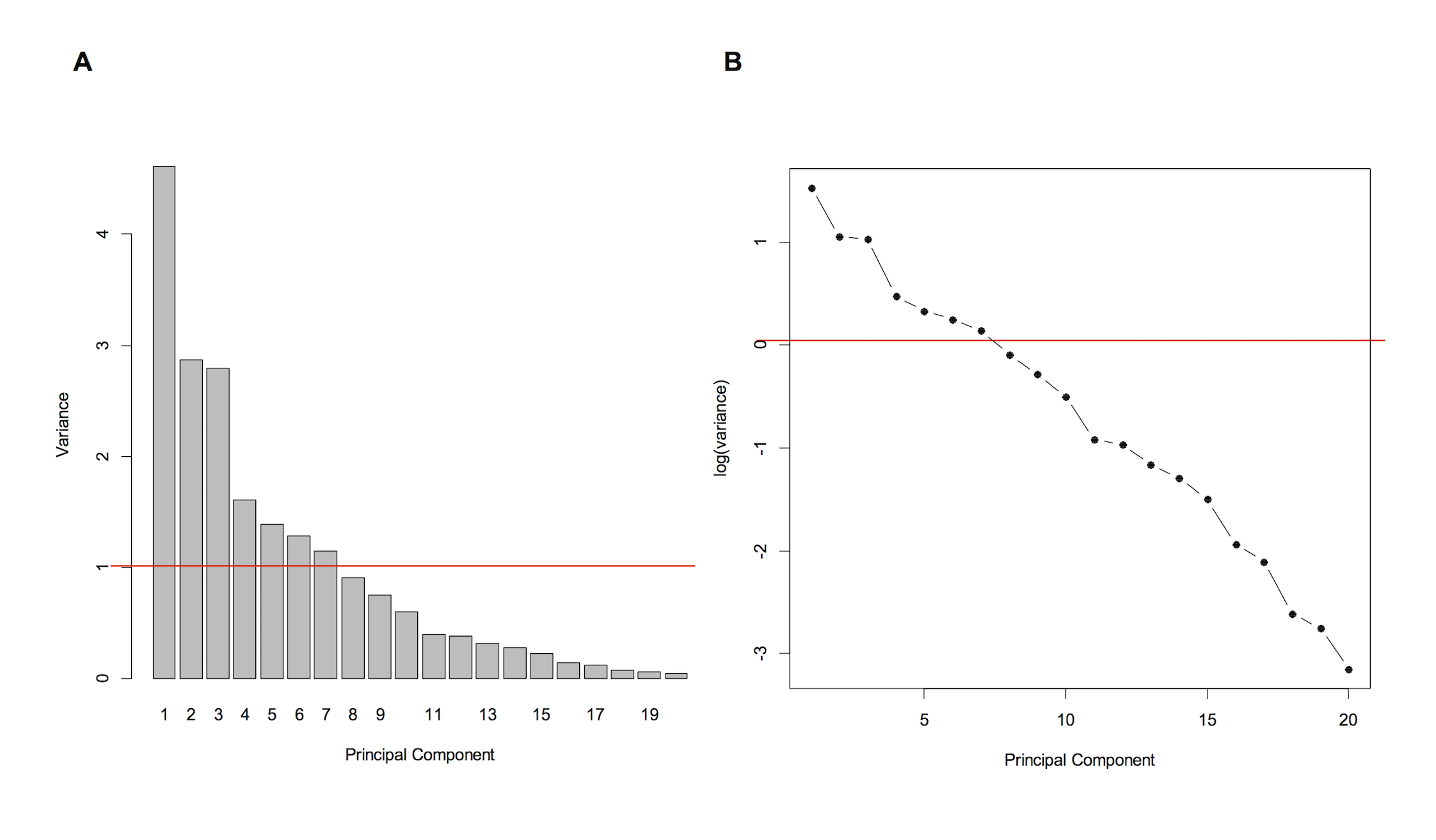

Supplement: S1 Fig — (A) Relative variance and (B) log absolute variance captured by each principal component. Red lines indicate truncation after the 7 leading principal components, which capture most of the variance and are most readily interpretable. (TIF) [file pcbi.1004185.s001.tif]

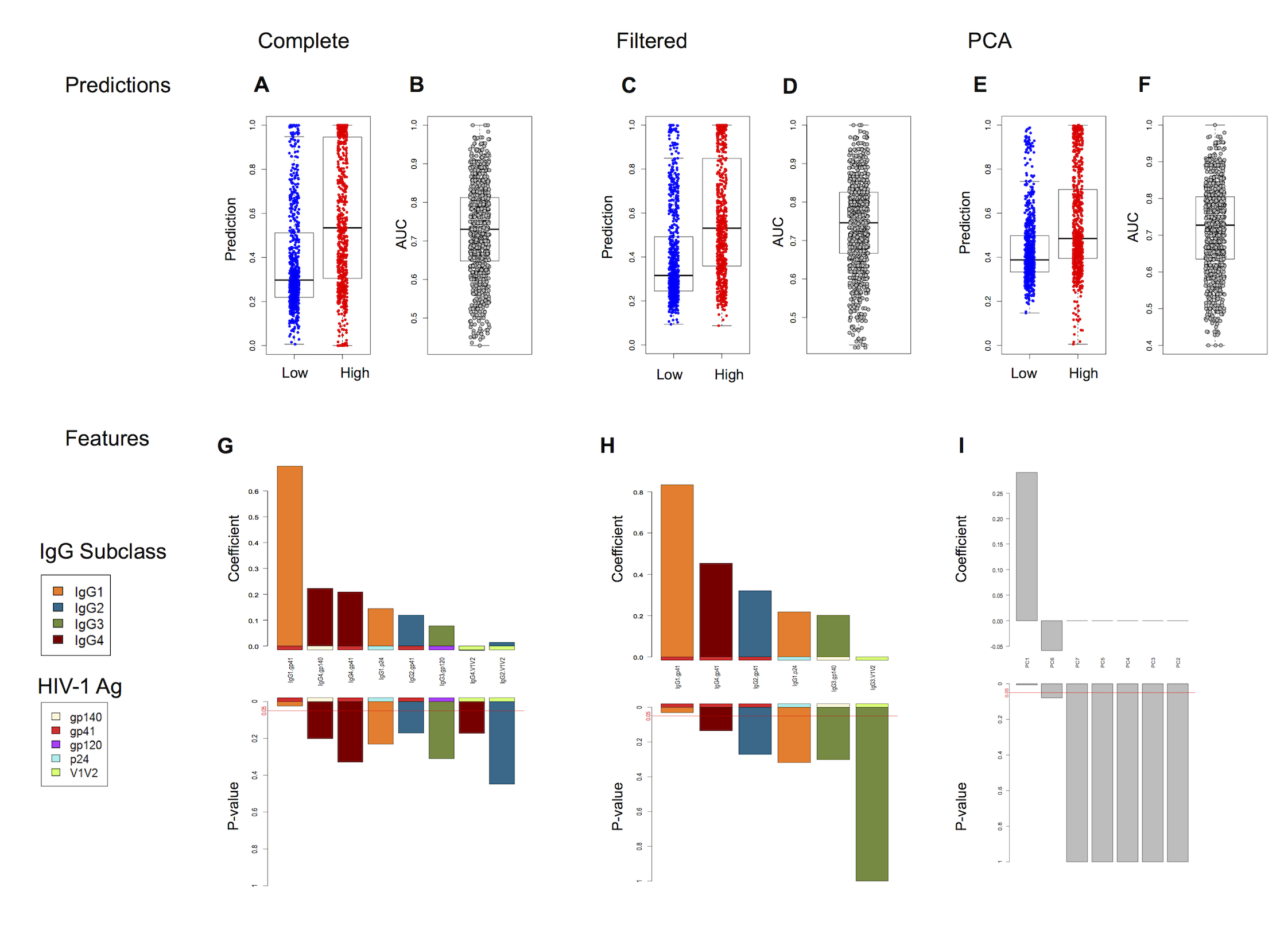

Supplement: S2 Fig — (A-F) Prediction results by 200-replicate five-fold cross-validation, illustrating PLR values (>0.5 predicted high ADCP; <0.5 predicted low) for one replicate (A,C,E) and providing area under the ROC curve (AUC) over all 200 replicates (B,D,F). Box & whisker plots show the median (thick center line), upper and lower quartiles (box), and 1.5 times the interquartile range (whiskers); all points are also plotted in a jittered stripchart. Colors for the classification examples indicate high (red) and low (blue) observed ADCP. (G-I) Coefficients and p-values of the features for a model trained on all subjects. Different input features were used in classification: (A,B,G) the complete set; (C,D,H) the filtered set; (E,F,I) the principal components. Colors for the feature coefficients indicate antibody subclass and antigen-specificity. For convenience, a red line is drawn at p = 0.05. (TIF) [file pcbi.1004185.s002.tif]

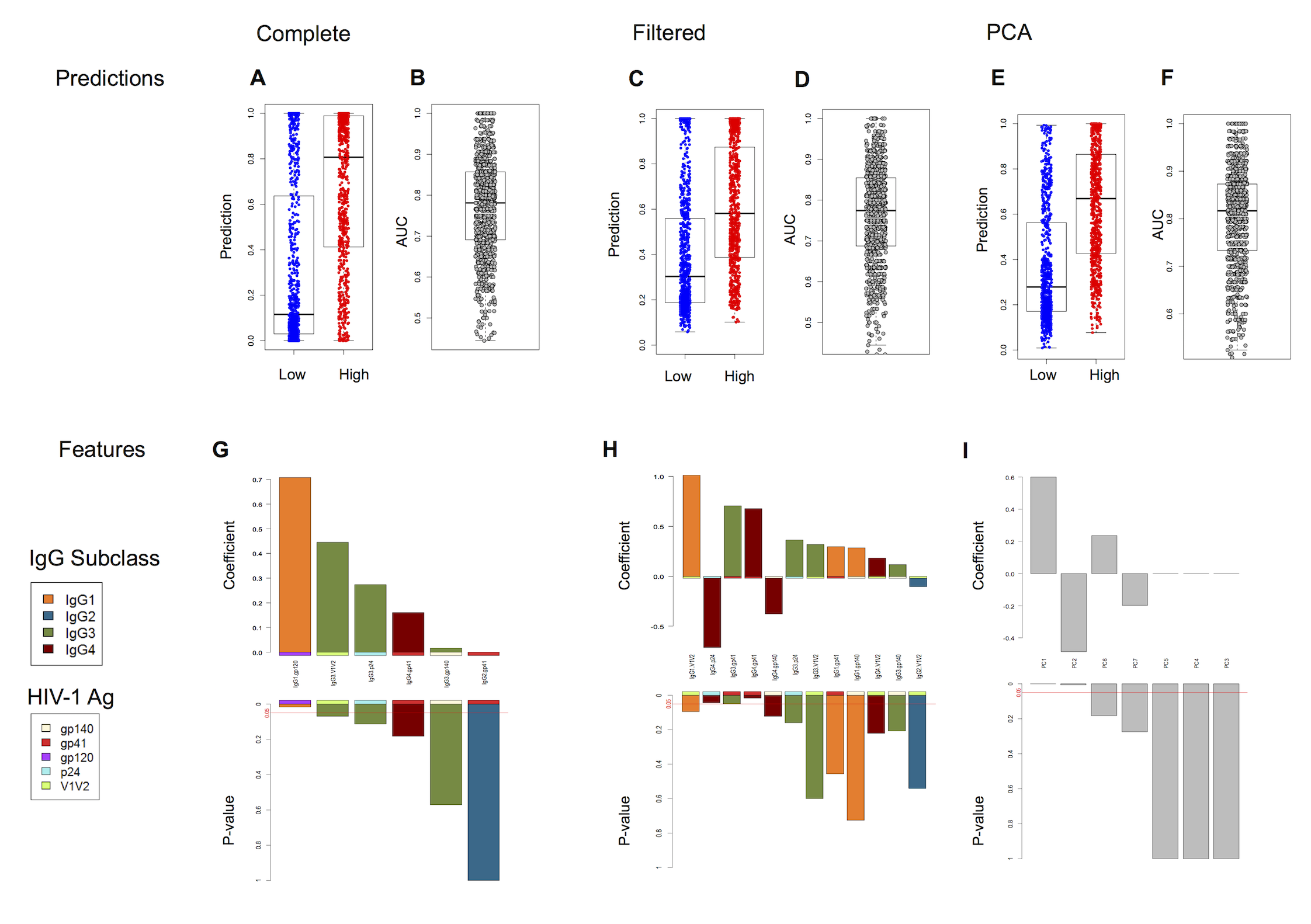

Supplement: S3 Fig — (A-F) Prediction results by 200-replicate five-fold cross-validation, illustrating PLR values (>0.5 predicted high ADCP; <0.5 predicted low) for one replicate (A,C,E) and providing area under the ROC curve (AUC) over all 200 replicates (B,D,F). Box & whisker plots show the median (thick center line), upper and lower quartiles (box), and 1.5 times the interquartile range (whiskers); all points are also plotted in a jittered stripchart. Colors for the classification examples indicate high (red) and low (blue) observed ADCP. (G-I) Coefficients and p-values of the features for a model trained on all subjects. Different input features were used in classification: (A,B,G) the complete set; (C,D,H) the filtered set; (E,F,I) the principal components. Colors for the feature coefficients indicate antibody subclass and antigen-specificity. For convenience, a red line is drawn at p = 0.05. (TIF) [file pcbi.1004185.s003.tif]

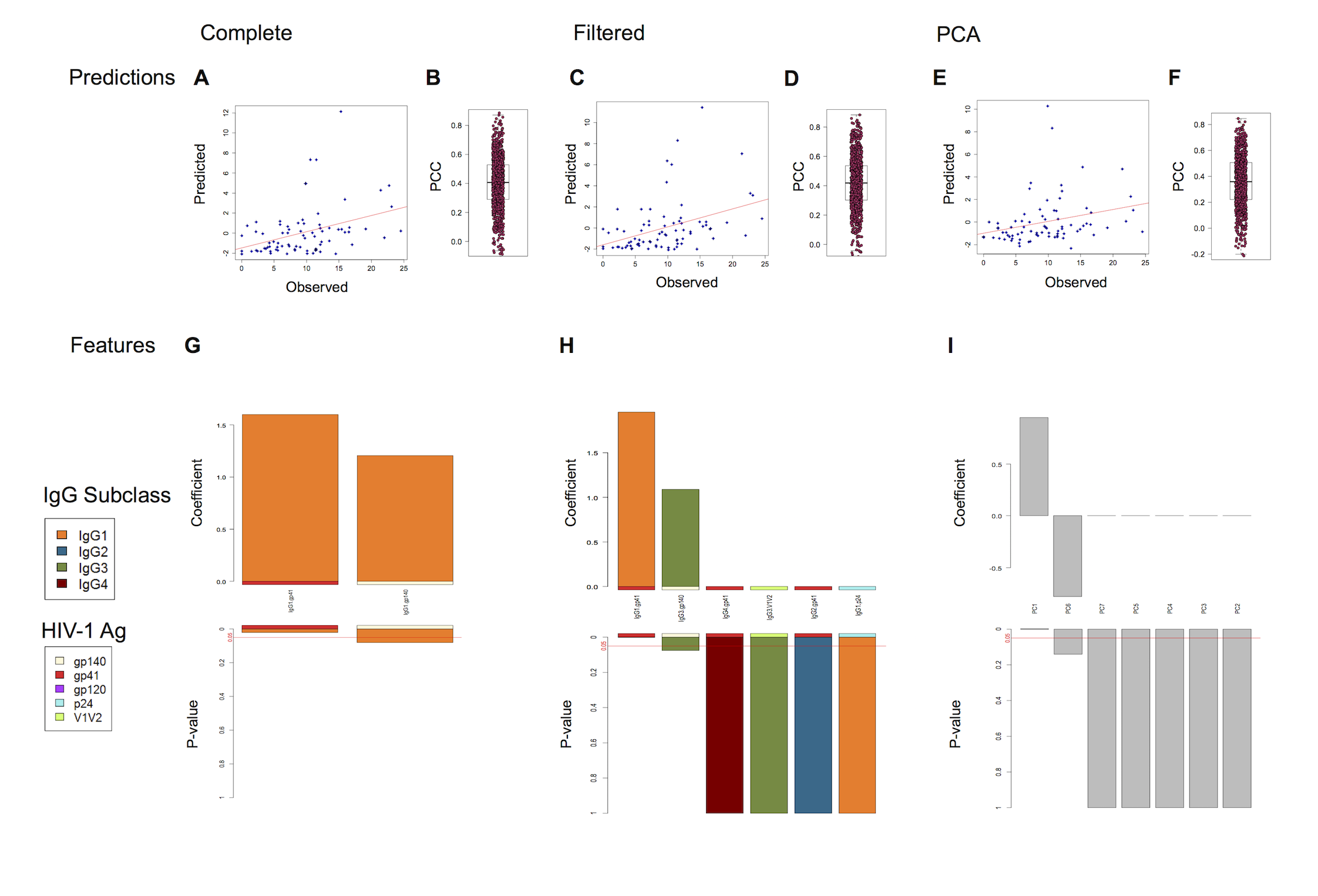

Supplement: S4 Fig — (A-F) Representative regression scatterplot based on leave-one-out cross-validation (A,C,E), and PCCs for 200-replicate five-fold cross-validation (B,D,F). (G-I) Coefficients and p-values of the features for a model trained on all subjects. Different input features were used: (A,B<G) the complete set; (C,D,H) the filtered set; (E,F,I) the principal components. Box & whisker plots show the median (thick center line), upper and lower quartiles (box), and 1.5 times the interquartile range (whiskers); all points are also plotted in a jittered stripchart. Colors for the feature coefficients indicate antibody subclass and antigen-specificity. (TIF) [file pcbi.1004185.s004.tif]

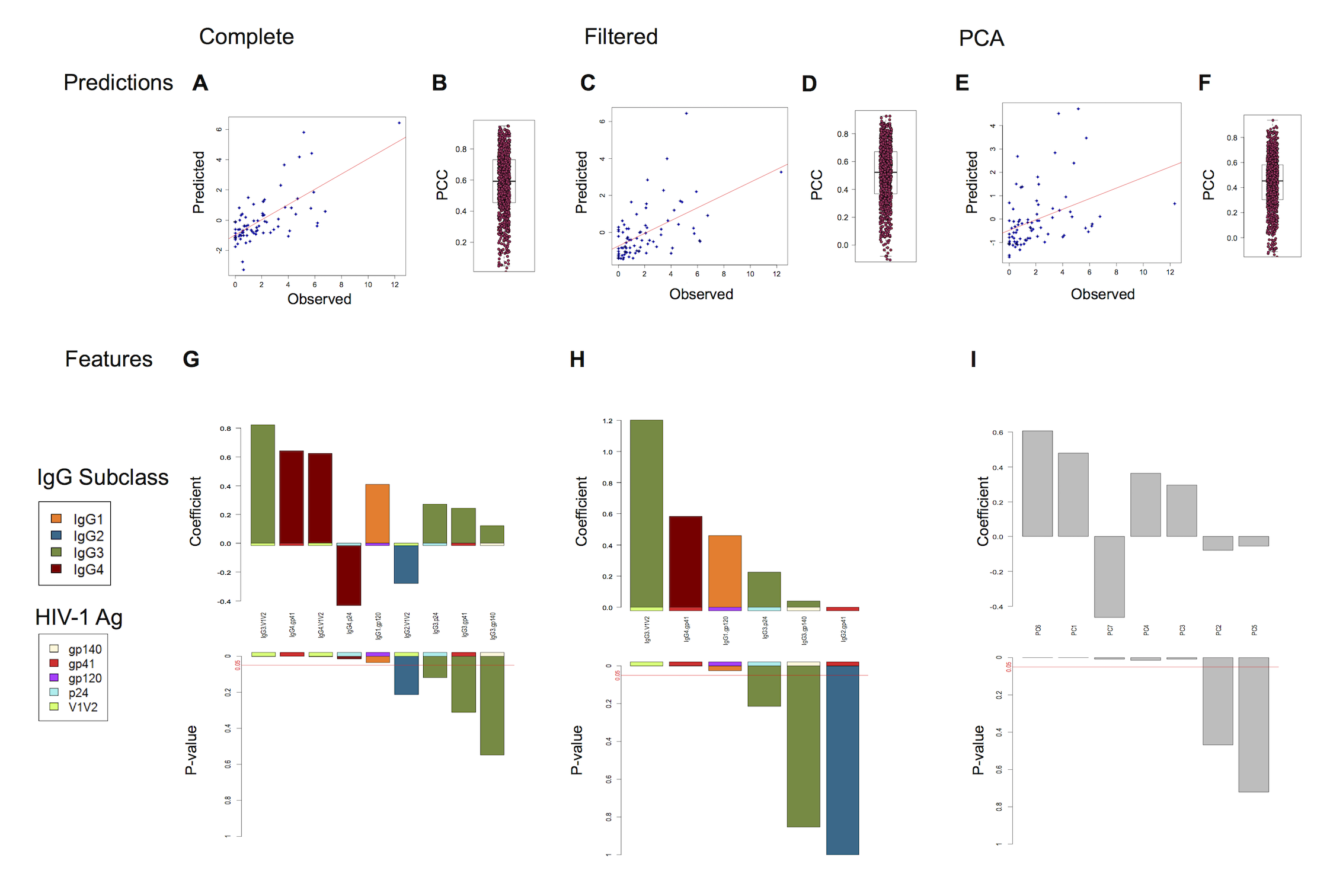

Supplement: S5 Fig — (A-F) Representative regression scatterplot based on leave-one-out cross-validation (A,C,E), and PCCs for 200-replicate five-fold cross-validation (B,D,F). (G-I) Coefficients and p-values of the features for a model trained on all subjects. Different input features were used: (A,B,G) the complete set; (C,D,H) the filtered set; (E,F,I) the principal components. Box & whisker plots show the median (thick center line), upper and lower quartiles (box), and 1.5 times the interquartile range (whiskers); all points are also plotted in a jittered stripchart. Colors for the feature coefficients indicate antibody subclass and antigen-specificity. (TIF) [file pcbi.1004185.s005.tif]
